# Mitochondrial respiration and redox coupling in articular chondrocytes

**DOI:** 10.1186/s13075-015-0566-9

**Published:** 2015-03-10

**Authors:** Rachel S Lane, Yao Fu, Satoshi Matsuzaki, Michael Kinter, Kenneth M Humphries, Timothy M Griffin

**Affiliations:** Free Radical Biology and Aging Program, Oklahoma Medical Research Foundation, MS 21, 825 NE 13th Street, Oklahoma City, OK 73104 USA; Department of Biochemistry and Molecular Biology, University of Oklahoma Health Sciences Center, 940 Stanton L. Young Blvd., BMSB 853, Oklahoma City, OK 73104 USA; Department of Geriatric Medicine, Reynolds Oklahoma Center on Aging, University of Oklahoma Health Sciences Center, 975 NE 10th Street, BRC-1303, Oklahoma City, OK 73104 USA

## Abstract

**Introduction:**

Chondrocytes rely primarily on glycolysis to meet cellular energy needs, but recent studies implicate impaired mitochondrial function in osteoarthritis (OA) pathogenesis. Our objectives were to investigate the ability of chondrocytes to upregulate mitochondrial respiration when challenged with a nutrient stress and determine the effect on mediators of chondrocyte oxidative homeostasis.

**Methods:**

Primary bovine chondrocytes were isolated and cultured in alginate beads. Mitochondrial respiration was stimulated by culturing cells with galactose-supplemented media for a period of 1 or 5 days. Metabolic flexibility was assessed by measuring metabolite and enzymatic biomarkers of glycolytic and mitochondrial metabolism. Oxidative homeostasis was assessed by measuring (1) cellular glutathione content and redox homeostasis, (2) rates of nitric oxide and superoxide production, and (3) the abundance and activity of cellular anti-oxidant proteins, especially the mitochondrial isoform of superoxide dismutase (SOD2). The regulatory role of hypoxia-inducible factor 2α (HIF-2α) in mediating the metabolic and redox responses was evaluated by chemical stabilization with cobalt chloride (CoCl_2_).

**Results:**

After 5 days of galactose culture, lactate production and lactate dehydrogenase activity were reduced by 92% (*P* <0.0001) and 28% (*P* = 0.051), respectively. Conversely, basal oxygen consumption increased 35% (*P* = 0.042) without increasing mitochondrial content. Glutathione redox homeostasis was unaffected by galactose culture. However, the production of nitric oxide and superoxide and the expression and activity of SOD2 were significantly reduced after 5 days in galactose culture. Nuclear protein expression and gene expression of HIF-2α, a transcription factor for SOD2, were significantly downregulated (more than twofold; *P* <0.05) with galactose culture. CoCl_2_-mediated stabilization of HIF-2α during the initial galactose response phase attenuated the reduction in SOD2 (*P* = 0.028) and increased cell death (*P* = 0.003).

**Conclusions:**

Chondrocyte metabolic flexibility promotes cell survival during a nutrient stress by upregulating mitochondrial respiration and reducing the rate of reactive nitrogen and oxygen species production. These changes are coupled to a substantial reduction in the expression and activity of the mitochondrial anti-oxidant SOD2 and its pro-catabolic transcription factor HIF-2α, suggesting that an improved understanding of physiologic triggers of chondrocyte metabolic flexibility may provide new insight into the etiology of OA.

**Electronic supplementary material:**

The online version of this article (doi:10.1186/s13075-015-0566-9) contains supplementary material, which is available to authorized users.

## Introduction

The avascular environment of articular cartilage is generally thought to restrict chondrocyte metabolism to relatively low rates of anaerobic glycolysis due to limits in the rate of oxygen and nutrient diffusion from the synovial fluid, particularly in the middle and deep cartilage zones [1-3]. In addition, the relatively low mitochondrial content and slow rates of respiration in chondrocytes may be considered adaptive for minimizing oxidative damage in long-lived post-mitotic cells [4]. These metabolic characteristics, however, do not appear to be wholly derived from the unique avascular cartilage environment and slow turnover of cells as they are also shared by mesenchymal stem cells (MSCs) [5]. MSCs are resistant to exposure to hypoxia or inhibition of mitochondrial respiration due to the strong reliance on anaerobic glycolysis for ATP production [5]. Thus, unlike many cells derived from MSCs that upregulate mitochondrial respiration during differentiation, chondrocytes appear to maintain a more undifferentiated MSC-like metabolic state [6].

The strong reliance on anaerobic glycolysis as the primary ATP-producing pathway of cartilage raises questions about the metabolic role of mitochondria in chondrocytes [7,8]. Mitochondrial respiration is not limited by oxygen availability, because even at normoxic oxygen concentrations chondrocytes continue to primarily use glycolysis for ATP production [8-10]. However, under anoxic conditions, chondrocytes reduce the rate of anaerobic glycolysis, demonstrating a negative Pasteur effect [10]. Recent studies suggest that the rate of glycolysis is dependent on at least a minimal flux of oxygen through the mitochondrial respiratory chain (MRC) to activate or stabilize glycolytic enzymes through MRC-derived reactive oxygen species (ROS) [11]. A better understanding of the relationship between chondrocyte metabolism and ROS production will help elucidate the functional role of mitochondria in chondrocyte metabolism and may provide insight into how mitochondrial dysfunction contributes to osteoarthritis (OA) disease pathology.

OA cartilage is characterized by multiple forms of oxidative modifications to lipids, proteins, and nucleic acids [12,13]. Impaired MRC activity is implicated as a source of pathologic ROS production leading to oxidative stress in OA [8,14,15]. In healthy cartilage, pro-inflammatory cytokines and nitric oxide inhibit the activity of complexes I and IV of the MRC, respectively, suggesting that increased mitochondrial-ROS production is a downstream consequence of cellular inflammation [16-18]. In addition to increased ROS production, mitochondria may be more susceptible to ROS damage with OA due to an impaired anti-oxidant system. In particular, SOD2, the mitochondrial isoform of superoxide dismutase, is downregulated in OA cartilage [8,19-21]. When SOD2 is silenced in healthy chondrocytes, cells accumulate malondialdehyde, a lipid peroxidation product [8]. In addition, mitochondria respire closer to their maximal capacity and increase mitochondrial proton leak [8]. This suggests that changes in the mitochondrial redox balance regulate mitochondrial respiration and perhaps overall cellular metabolism. Therefore, a better understanding of the relationship between cellular redox and metabolic flexibility in healthy chondrocytes may generate new insight into the role of altered metabolism in the pathogenesis of OA.

There were two goals of this study. First, we wanted to determine the capacity and mechanisms by which chondrocytes upregulate mitochondrial respiration in response to a nutrient stress. Mitochondrial metabolism is an efficient means of producing ATP when metabolic substrates are limiting, and under growth or repair conditions, insufficient MRC activity may lead to a depletion of cellular ATP levels [22]. Second, we wanted to determine the effect of upregulating MRC activity on chondrocyte redox balance. Chondrocyte metabolism undergoes dynamic changes in response to inflammatory and mechanical stressors [23-27]. Understanding how chondrocyte redox homeostasis is affected during changes in cellular metabolism independent of these additional stressors is important for identifying potential metabolic origins of oxidative stress in OA.

We stimulated chondrocyte MRC activity by replacing glucose with galactose in the cell culture media of healthy primary bovine chondrocytes. Galactose creates a nutrient stress by requiring additional energy to convert to glucose. In mammalian cells, replacing glucose with galactose as the sole sugar source in the culture media is an effective strategy for stimulating mitochondrial oxidative phosphorylation and evaluating mitochondrial disorders and drug toxicity [28-30]. Here, we show how a galactose-induced metabolic stress stimulates chondrocyte MRC activity and impacts mitochondrial redox regulation.

## Methods

### Cell culture

Bovine fetlock joints were purchased from a local slaughterhouse in accordance with a protocol approved from the Oklahoma Medical Research Foundation (OMRF) Institutional Animal Care and Use Committee. Joints were cleaned and cartilage was extracted for cellular isolation within 8 hours of death. Cartilage was incubated in 1,320 U Pronase (Calbiochem from EMD Millipore, Billerica, MA, USA) per mL low glucose Dulbecco’s modified Eagle’s medium (DMEM) supplemented with kanamycin (100 μg/mL), gentamycin (150 μg/mL), non-essential amino acids, HEPES (10 mM), 5% fetal bovine serum, and penicillin-streptomycin (50 U/mL) for 1 hour (Gibco brand media reagents from Life Technologies, Carlsbad, CA, USA). Pronase-enriched media was then replaced with 0.3% collagenase, type 2 (Worthington Biochemical Corporation, Lakewood, NJ, USA), in low-glucose DMEM culture media containing non-essential amino acids, HEPES (10 mM), 5% fetal bovine serum, and 100 U/mL penicillin-streptomycin and incubated overnight. Cells were strained through a 70-μm filter, counted, and assessed for viability by using trypan blue exclusion and a Cellometer AutoT4 cell counter (Nexcelom Bioscience, Lawrence, MA, USA). Finally, cells were re-suspeneded in 2.0% alginate 150 mM sodium chloride solution (pH 7.4) at 4 × 10^6^ cells/mL. The cell solution was carefully pipetted into a 102 mM calcium chloride solution (pH 7.4) to encapsulate the cells in alginate beads. Beads were cultured in 6 mM glucose culture media or no-glucose, no pyruvate culture media enriched with 6 mM galactose for a period of up to 5 days. For 1-day galactose experiments, cells were maintained in glucose-supplemented media for 4 to 5 days prior to replacing with fresh glucose or galactose media, thereby minimizing differences in total culture duration. To compare the difference between galactose treatment and glycolysis inhibition on cell viability, we also cultured cells in 6 mM 2-deoxy-D-glucose (Sigma-Aldrich, St. Louis, MO, USA), a glucose analog that inhibits glycolysis. To quantify HIF-2α nuclear expression, 200 μM cobalt chloride (CoCl_2_) (ACROS Organics from Thermo Fisher Scientific, Waltham, MA, USA) was added to the media 1 day prior to harvest [31]. Cells were digested out of alginate with 55 mM sodium citrate (pH 6) and tested for viability as previously described.

Cells were re-suspended in the following concentrations and buffers according to the following analyses: (1) cell respiration: 2 × 10^6^ cells/mL phosphate-buffered saline (PBS) (pH 7.4); (2) enzyme activity: 10^6^ cells/mL 1.0 mM MOPS/10 mM EDTA (pH 7.4); (3) mRNA quantification: 10^7^ cells/mL TRIzol; and (4) Western blot: 10^7^ cells/mL RIPA with 0.1% NP40 (pH 7.4). Protein concentration for cell lysates was quantified by using the Pierce BCA protein assay (Thermo Fisher Scientific).

### Cell respiration and mitochondrial staining

Chondrocyte respiration was measured by using a Clark-style oxygen electrode (Instech, Plymouth Meeting, PA, USA) in a temperature-regulated chamber set to 37°C (Hansatech Instruments Ltd, Norfolk, UK). The starting amount of molecular oxygen in the 0.6-mL electrode chamber was based on the assumption that 213 nmol/mL of molecular oxygen is dissolved at atmospheric pressure and 37°C. Basal respiration was measured as the average rate of unstimulated oxygen consumption. Maximal respiration was determined after stimulation with 0.8 μM FCCP, an electron transport chain uncoupler. Mitochondrial-specific oxygen consumption was determined by addition of cyanide. To evaluate mitochondrial content, cells were stained with Mitotracker Green FM (Molecular Probes from Life Technologies) and fluorescent intensity was measured by using a FACSCalibur flow cytometer (BD Biosciences, San Jose, CA, USA). Data were analyzed by comparing the mean fluorescent intensity of glucose versus galactose-cultured cells by using FlowJo software. We also assessed mitochondrial content by using selected reaction monitoring (SRM) mass spectrometry to quantify the abundance of two mitochondrial reference proteins, ATP5B and VDAC1, as described in detail further below.

### Metabolic and redox biomarkers and enzymatic activities

#### Lactate dehydrogenase activity

Lactate dehydrogenase (LDH) activity was measured spectrophotometrically as the rotenone-independent oxidation of 205 μM NADH to NAD^+^ (Agilent 8452A; Agilent Technologies, Santa Clara, CA, USA) by monitoring the decrease in A_340nm_ in the presence of 10 μg protein and 1.5 mM pyruvate (Sigma-Aldrich) in 25 mM MOPS buffer (pH 7.4). Activity was determined to be LDH specific by using 25 mM of the competitive inhibitor, oxamate (Sigma-Aldrich).

#### Superoxide dismutase activity

Total (tSOD) and manganese-specific (SOD2) SOD activity was determined spectrophotometrically (Sunrise™; Tecan US, Morrisville, NC, USA) in accordance with the instructions of the manufacturer (Cayman Chemicals Company, Ann Arbor, MI, USA).

#### Glucose and lactate measurement

Conditioned media was collected for measurement of glucose and lactate concentration by using a YSI 2300 STAT Plus Glucose and Lactate Analyzer (Yellow Springs Instruments, Yellow Springs, OH, USA). Conditioned media samples were standardized to plate-matched non-conditioned media blanks.

#### Nitric oxide measurement

Total nitrate and nitrite (NO_x_) secretion into the media was measured by using the Greiss reaction as previously described [32].

#### Glutathione assessment

Oxidized and reduced glutathione were measured spectrophotometrically (Tecan US) by using an enzymatic recycling method to quantify the production of 5-thio-2-nitrobenzoic acid (TNB) generated from the reaction of reduced glutathione (GSH) with 5′-5′ dithio-*bis*-2 (nitrobenzoic acid) (DTNB) in accordance with the instructions of the manufacturer (Cayman Chemicals Company).

#### NAD^+^/NADH

The intracellular ratio of NAD^+^ to NADH in cell lysates was measured by using an enzyme recycling reaction to quantitate NADH absorbance in accordance with the instructions of the manufacturer (BioVision, Inc., Milpitas, CA, USA).

#### Energy charge

High-performance liquid chromatography (Shimadzu LC-20A High Precision Binary Gradient HPLC system; Shimadzu, Kyoto, Japan) and a UV/VIS diode array spectrometer were used to resolve and detect AMP, ADP, and ATP. The mobile phase consisted of 100 mM KH_2_PO_4_ and 1.0 mM tetrabutylammonium sulfate (TBAS) at pH 6.0 (buffer A) and CH_3_CN (buffer B) with a flow rate of 1.0 mL/minute over an Eclipse Plus C18 column with 5 μM diameter beads, 4.6 × 150 mM in length (Agilent Technologies). Adenylate nucleotides were separated by using the following step-wise gradients of buffer A/B: 96%/4% for 5 minutes, 85%/15% for 10 minutes, and 96%/4% for 5 minutes. Concentrations of ATP, ADP, and AMP were detected by absorption at 254 nm and quantified on the basis of the integrated area of standards. Energy charge was calculated by using the equation: ([ATP] + 0.5[ADP])/ ([ATP] + [ADP] + [AMP]).

### Cellular free radical production

Superoxide production was assessed by electron paramagnetic resonance (EPR) spin-trapping using a cyclic hydroxylamine, CMH (1-hydroxy-3-methoxycarbonyl-2,2,5,5-tetramethylpyrrolidine) [33]. Chondrocytes were isolated as previously described and cultured in monolayer for 3 days in glucose-supplemented culture media at 4.0 × 10^4^ cells per well in a 48-well plate. Culture media were then replaced with either glucose- or galactose-supplemented DMEM culture media for 24 hours. After washing with PBS, adherent cells were incubated with 500 μM CMH in the presence of 1 mM EDTA and 50 μM DTPA in PBS at 37°C for 15 minutes. Reacted spin-traps were immediately snap-frozen in LN_2_ after the incubation period until the EPR measurement. High-density monolayer culture was required in place of alginate bead culture to improve the rapid intra- and extra-cellular equilibration of the spin-trap and thereby maximize the signal to noise ratio.

The EPR spectra were obtained by using a Bruker EMX spectrometer (Bruker Corporation, Billerica, MA, USA) operating at X-band (approximately 9.78 GHz) with a 100 kHz modulation frequency and ER 41225SHQ high-sensitivity cavity. Typical settings for the spectrometer are microwave power, 6.325 mW; modulation amplitude, 1.5 G; scan range, 50 G; time constant, 82 ms. Thawed sample mixtures were transferred immediately to a quartz flat-cell for the EPR determination. All of the EPR experiments were performed at room temperature.

### RNA extraction, reverse transcription, and quantitative real-time polymerase chain reaction

Immediately after chondrocyte digestion from alginate beads, RNA was stabilized by using TRIzol in accordance with the instructions of the manufacturer (Life Technologies). A Qiagen First Strand cDNA kit (Qiagen, Hilden, Germany) was used to convert mRNA to cDNA in accordance with the instructions of the manufacturer. Primers for *EPAS1*, *SOD2*, *SOD1*, *CAT*, *COL2*, *NOS2*, *ACAN*, *PTGS2*, *MMP13*, *ADAMTS4*, *HIF1*, *TFAM*, *PGC1A*, *RLPLO*, *GAPDH*, *B-Actin*, and *B2M* were purchased from Qiagen’s validated RT^2^ qPCR Primer Assays to quantify gene expression. A Bio-Rad CFX96 Real-Time Detection system (Bio-Rad Laboratories, Hercules, CA, USA) was used for amplification and quantification of amplicons. Target genes were standardized to the geometric mean of four housekeeping genes (*RLPLO*, *GAPDH*, *B-Actin*, and *B2M*). Results were expressed as standardized gene expression (2^−ΔCt^) or gene expression of the galactose-treated sample normalized to the animal-matched glucose control.

### Protein extraction and Western blot analysis

Cell lysates were centrifuged at 14,000 *g* for 10 minutes to separate cytosolic and nuclear proteins. The nucleic fraction was re-suspended in SDS running buffer, sonicated, and centrifuged again at 14,000 *g* for 10 minutes for further clarification. Protein concentrations were determined by Bradford assay and equalized between conditions, separated on a 4% to 12% NuPAGE Bis-Tris gels (Life Technologies), and transferred onto a polyvinylidene difluoride (PVDF) or nitrocellulose membranes. The following proteins were detected by using experimentally determined antibody concentrations: succinate dehydrogenase subunit A (SDHA) (1:500; Santa Cruz Biotechnology, Inc., Dallas, TX, USA), superoxide dismutase 1 (SOD1, 1:10,000; Santa Cruz Biotechnology, Inc.), superoxide dismutase 2 (SOD2) (1:10,000, Santa Cruz Biotechnology, Inc.), hypoxia-inducible factor-2alpha (HIF-2α) (1:500; LifeSpan BioSciences, Inc., Seattle, WA, USA), Lamin B1 (1:1,000; Santa Cruz Biotechnology, Inc.), and actin conjugated to horseradish peroxidase (actin-HRP) (1:3,000; Santa Cruz Biotechnology, Inc.). Expression was quantified by using ImageJ software. To minimize the contribution of inter-animal variation to reported outcomes, glucose and galactose protein expression densities were normalized to the total density on an animal-to-animal basis and then averaged between animals. Proteins of interest were standardized to Actin or Lamin B1 for extra-nuclear and nuclear proteins, respectively.

### Quantitative mass spectrometry analysis

SRM mass spectrometry was used to quantify anti-oxidant protein expression as previously described [34]. Briefly, 3 pmol of equine serum albumin (ESA) was added to each 20-μg sample of chondrocyte protein as an internal standard. The mixture was precipitated by acetone and suspended in Laemmli loading buffer. Samples were run in an SDS-PAGE gel to a distance of 1.5 cm. The entire lane was cut for each sample and divided into 1-mm^3^ pieces, reduced with DTT, alkylated with iodoacetamide, and digested with trypsin. The peptides produced were extracted from the gel by 50% methanol with 10% formic acid. The extract was evaporated to dryness and dissolved in 150 μL of 1% acetic acid for analysis. Samples were analyzed by using a TSQ Vantage triple quadrupole mass spectrometer (Thermo Fisher Scientific), operated in the SRM mode with a splitless nanoflow HPLC system (Eksigent, Dublin, CA, USA). Samples (10 μL) were injected onto a 10 cm × 75 μm C18 capillary column. Data were processed by using Pinpoint to find and integrate the correct peptide chromatographic peaks. To quantify protein expression, the relative abundance of each protein was first normalized to the ESA internal standard and then normalized to the geometric mean of four stable cellular reference proteins: glyceraldehyde-3-phosphate dehydrogenase (GAPDH), peptidyl-prolyl isomerase A (PPIA), ribosomal protein S27a (RPS27A), and vimentin (VIM).

### Statistical analyses

Statistical significance of galactose or CoCl_2_ treatment was determined by paired two- or one-tailed Student’s *t* tests, as appropriate. The effect of culture duration in addition to galactose treatment was determined by using a two-way analysis of variance with repeated measures for animal matching and Holm-Sidak’s multiple comparisons *post hoc* analysis. Significance was determined as a *P* value of less than 0.05. Analyses were carried out by using Prism 6 (GraphPad Software Inc., San Diego, CA, USA). Results are reported as the mean ± standard error of the mean for at least three individual animals as specified in the figure legends.

## Results

### Effect of galactose culture on chondrocyte metabolism

**Figure 1:**
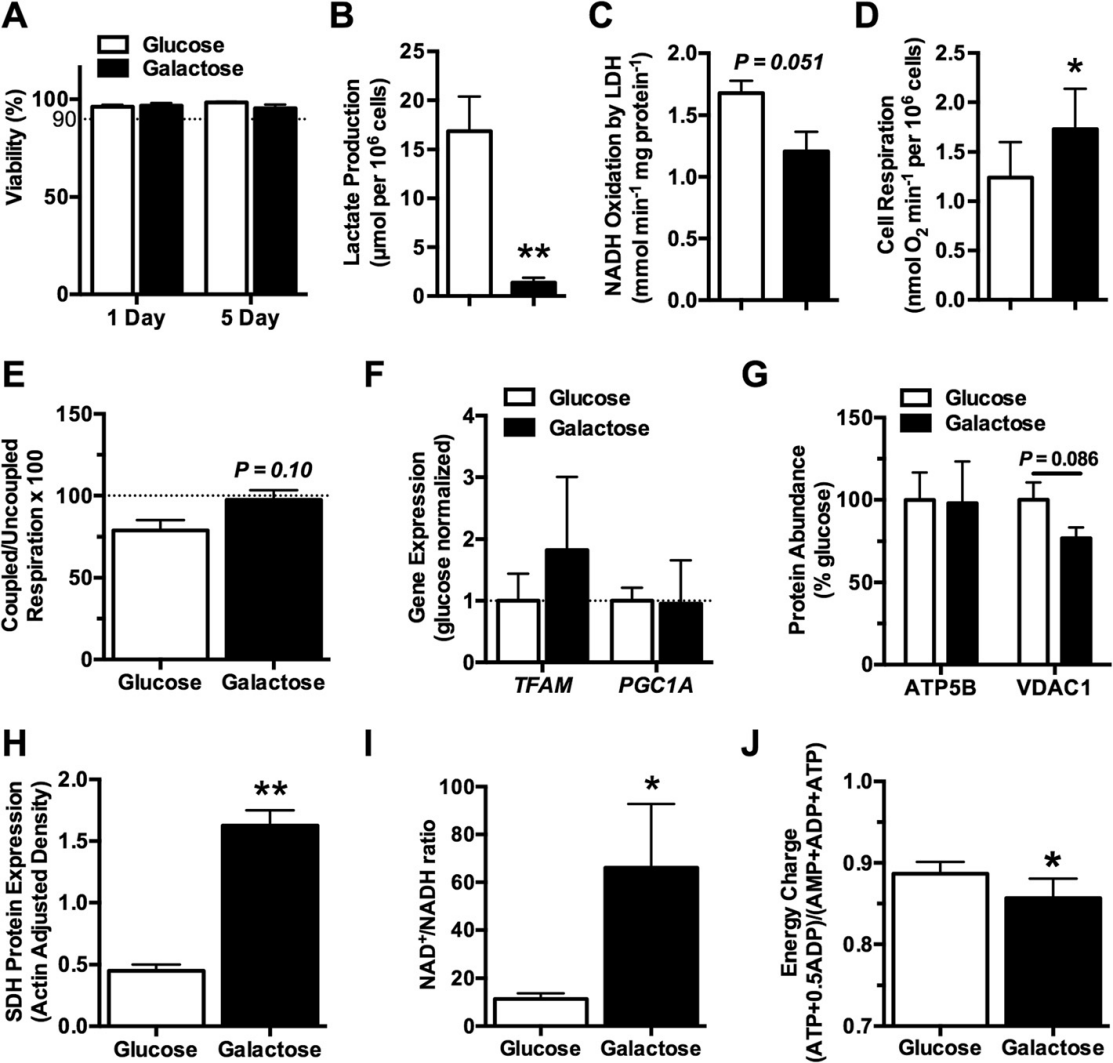
**Replacing glucose with galactose reduces glycolysis and upregulates mitochondrial respiration. (A)** Cell viability was not altered by 1 or 5 days of galactose culture (n = 6). Five days of galactose culture **(B)** significantly reduced lactate production (n = 6) and **(C)** trended toward a decrease in lactate dehydrogenase (LDH) activity (n = 4), indicating a reduction in non-oxidative glycolytic flux. The reduction in glycolysis after 5 days in galactose culture was offset by **(D)** an increase in the basal rate of cellular oxygen consumption (n = 6), which was associated with a near maximal rate of oxygen consumption, as indicated by **(E)** the ratio of coupled to uncoupled respiration approaching 100 (n = 4). The increase in mitochondrial respiration did not correspond to **(F)** an increase in the expression of genetic mediators of mitochondrial biogenesis (TFAM and PGC1A) after 1 day of galactose culture (n = 4) or an **(G)** increased abundance of mitochondrial proteins (ATP5B and VDAC1) after 5 days in galactose culture (n = 3). **(H)** However, 5 days of galactose culture significantly increased the expression of the mitochondrial electron transport chain and Krebs cycle enzyme succinate dehydrogenase (SDH) (n = 4). These metabolic changes were not able to maintain cellular metabolic homeostasis after 5 days of galactose culture, as indicated by **(I)** an increased ratio of NAD^+^ to NADH (n = 4) and **(J)** a decrease in the cellular energy charge (n = 3). Bars represent mean ± standard error of the mean. *P <0.05 and **P <0.01 between glucose and galactose.

Culturing chondrocytes in either glucose- or galactose-supplemented media for 1 or 5 days did not alter cell viability (Figure 1A). However, galactose culture did significantly alter chondrocyte metabolism. After 1 day in galactose culture, lactate production decreased 54%, from 17.3 to 8.0 μmol per 10^6^ cells (*P* <0.0001). After 5 days of galactose culture, both lactate production and maximal LDH activity were substantially reduced. Lactate production decreased by 92% (*P* <0.0001; Figure 1B), and LDH activity was reduced by 28% (*P* = 0.051; Figure 1C). These results are consistent with a substantial reduction in glycolytic flux and a reduced reliance on glycolysis for cellular ATP production. Galactose treatment, however, was not equivalent to complete glycolytic inhibition. Culturing chondrocytes for 1 day in 2-deoxy-D-glucose, a glucose analog that inhibits glycolysis, caused a modest 8% increase in cell death compared with galactose culture.

Chondrocytes responded to the galactose-induced reduction in glycolytic flux by increasing mitochondrial respiration. After 1 day in galactose culture, basal oxygen consumption increased 16% (*P* = 0.24), and by 5 days, basal oxygen consumption increased 40% (*P* = 0.042; Figure 1D). The increase in oxygen consumption with galactose culture was associated with a trend for cells respiring at a higher percentage of their maximal capacity compared with glucose-cultured cells (*P* = 0.10; Figure 1E). Five days in galactose culture did not increase the uncoupled (that is, maximal) rate of oxygen consumption (3.35 versus 3.51 μmol O_2_ consumption mL^−1^ min^−1^ per 10^6^ cells in glucose versus galactose media, respectively; *P* = 0.40), suggesting that galactose culture did not increase mitochondrial content. Consistent with this, we found that 1 day of galactose culture did not increase the expression of mitochondrial biogenesis transcription factors *TFAM* and *PGC1A* (Figure 1 F). Furthermore, after 5 days in galactose culture, the abundance of two mitochondrial-associated proteins, ATP5B and VDAC1, was not significantly altered (Figure 1G). However, the average intensity of Mitotracker staining showed a trend for an increase of approximately 50% between 1 and 5 days of galactose culture (*P* = 0.052). In addition, protein levels of succinate dehydrogenase (SDH), a Krebs cycle enzyme and component of complex II of the MRC, increased after 5 days in galactose culture (*P* = 0.003; Figure 1H). Thus, the increase in basal oxygen consumption after galactose culture appears to be driven primarily by increased mitochondrial oxygen consumption and electron transport flux rather than increased mitochondrial content, although specific mitochondrial proteins, such as SDH, are increased.

We next assessed the metabolic redox and energy state of chondrocytes by measuring the NAD^+^/NADH ratio and the cellular energy charge, respectively. After 5 days in galactose culture, the NAD^+^/NADH ratio was increased by approximately 50% compared with glucose-cultured cells, indicating a more oxidative cellular metabolic environment (*P* = 0.029; Figure 1I). To determine whether 5 days of galactose culture induced a sustained metabolic stress, we measured AMP, ADP, and ATP to calculate the energy charge for each culture condition. The cellular adenylate energy charge is tightly regulated and usually maintained at values between 0.88 and 0.92 [35,36]. After 5 days of galactose culture, the energy charge was reduced relative to the glucose culture condition (0.89 ± 0.01 versus 0.86 ± 0.02, glucose versus galactose; *P* = 0.048; Figure 1 J). Thus, replacing glucose with galactose as a carbohydrate source for 5 days induced a modest cellular energetic stress. Overall, these findings indicate that, in response to a nutritional energetic stress, chondrocytes upregulate mitochondrial metabolic pathways in an attempt to maintain energetic balance.

### Effect of galactose culture on redox balance and anti-oxidant function

**Figure 2:**
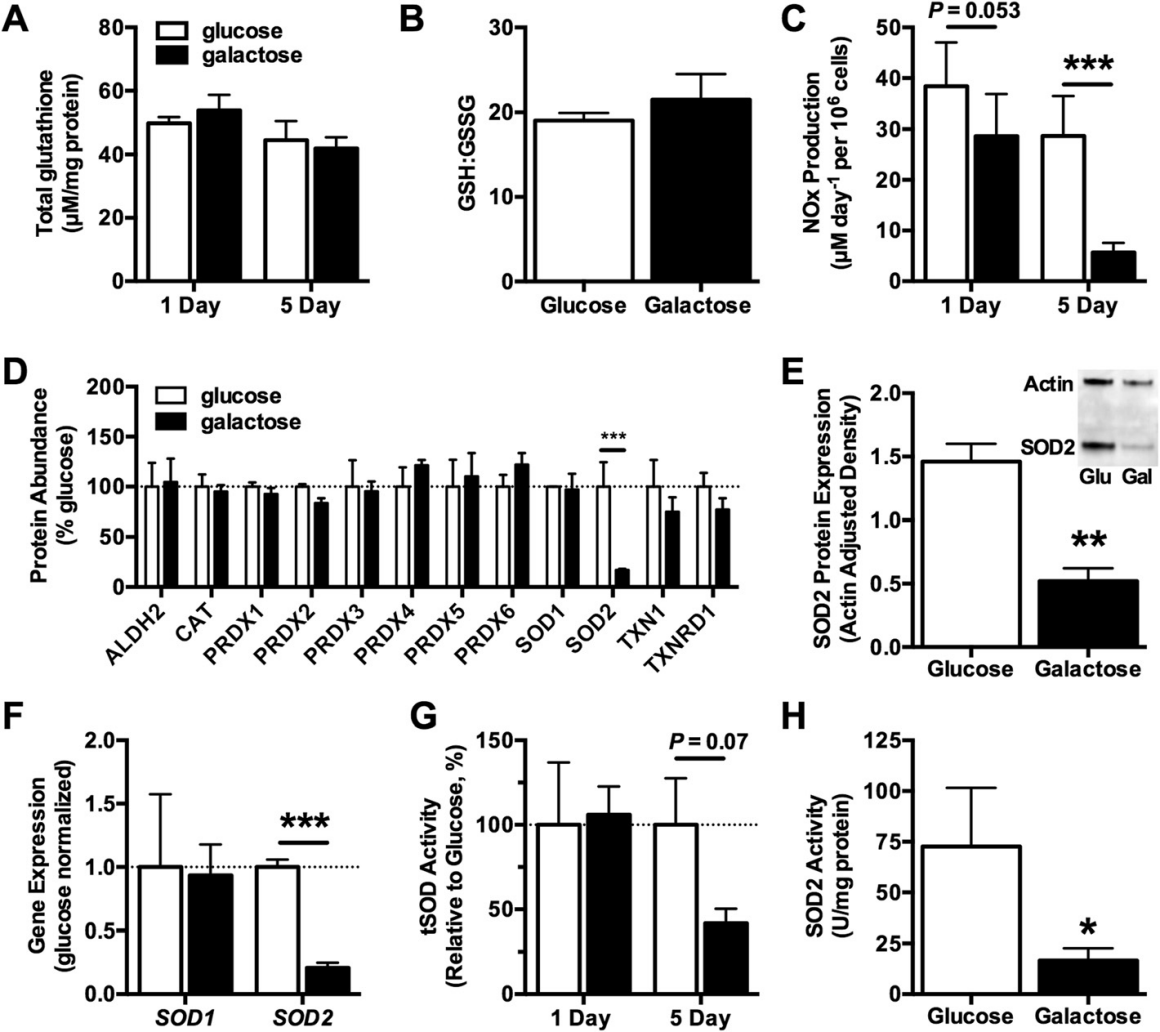
**Galactose treatment downregulates nitric oxide production and the mitochondrial anti-oxidant SOD2 without altering oxidative homeostasis. (A)** Cell glutathione content was not altered by 1 or 5 days of galactose culture (n = 4). **(B)** glutathione redox homeostasis was maintained after 5 days of galactose treatment (n = 4). **(C)** Five days of galactose culture significantly reduced nitrate and nitrite (NO_x_) release into the culture media (n = 13). **(D)** Out of a panel of 12 cytosolic and mitochondrial anti-oxidant proteins, 5 days of galactose culture selectively reduced the abundance of the mitochondrial SOD isoform, SOD2, as determined by selected reaction monitoring mass spectrometry (n = 3). **(E)** Western blot analysis further verified the reduction in SOD2 abundance (n = 5). **(F)** Real-time polymerase chain reaction analysis showed a significant reduction in gene expression of SOD2 (n = 5) but not the cytosolic SOD isoform, SOD1 (n = 4), after 5 days of galactose treatment. **(G)** Five days of galactose treatment reduced the total activity of superoxide dismutase (tSOD) (n = 7). The reduction in tSOD activity after 5 days in galactose culture was due primarily to a reduction in the activity of the mitochondrial SOD isoform, SOD2 (n = 7), **(H)** which paralleled the reduction in SOD2 protein expression. Bars represent mean ± standard error of the mean. *P <0.05, **P <0.01, and ***P <0.001 between glucose and galactose. Gal, galactose; Glu, glucose; GSH:GSSG, ratio of reduced to oxidized glutathione; SOD, superoxide dismutase.

We next investigated the effect of a shift toward increased mitochondrial respiration on pro- and anti-oxidant pathways affecting chondrocyte redox balance. One of the primary ways that cells maintain redox balance is through the synthesis of glutathione. Reduced glutathione (GSH) is a multi-faceted cellular anti-oxidant that directly reacts with free radicals, serves as a cofactor for glutathione peroxidase, and reverses oxidative modifications by reducing disulfide bonds [37]. Total glutathione levels remained consistent between glucose and galactose culture conditions (Figure 2A). In addition, the ratio of reduced to oxidized glutathione (GSH/GSSG), an indicator of cellular redox balance, was also consistent between day 5 glucose and galactose conditions (Figure 2B). We next investigated the basal production of nitric oxide by measuring the levels of nitrite and nitrate (NO_x_) released into the media. One day of galactose culture reduced NO_x_ levels by 25% (*P* = 0.053), and 5 days of galactose culture reduced NO_x_ release by 80% (*P* <0.001) compared with paired glucose controls (Figure 2C). Thus, although overall glutathione redox balance was unaffected by galactose, galactose-stimulated mitochondrial respiration significantly reduced the production of nitric oxide.

**Figure 3:**
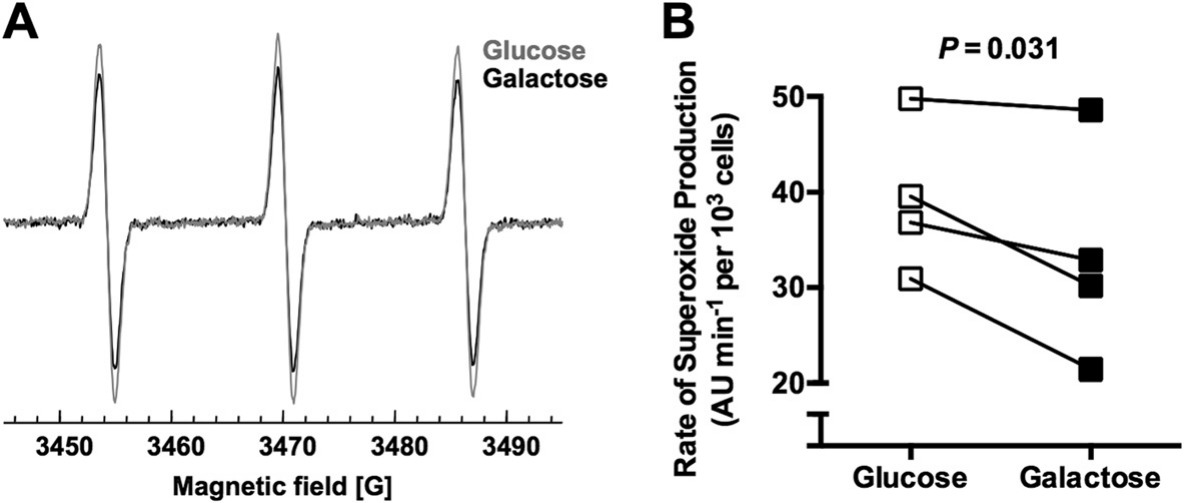
**Galactose-stimulated mitochondrial respiration reduces the rate of superoxide production. (A)** Representative raw spectra derived from electron paramagnetic resonance spectroscopy using the superoxide-specific cyclic hydroxylamine spin trap CMH (1-hydroxy-3-methoxycarbonyl-2,2,5,5-tetramethylpyrrolidine). The comparison of spectra from cells cultured with glucose or galactose for 1 day shows a reduction in spectral signal strength with galactose treatment, which is proportional to the rate of trapped superoxide. **(B)** The average rate of superoxide generation was significantly reduced after 1 day of galactose culture compared with pair-matched glucose-cultured samples (n = 4). AU, arbitrary units.

To further understand the effect of increased mitochondrial respiration on chondrocyte redox regulation, we quantified the abundance of 12 cellular anti-oxidant proteins by using SRM mass spectrometry after 5 days of glucose or galactose culture (Figure 2D). This analysis showed that stimulating mitochondrial respiration reduced the abundance of the mitochondrial isoform of superoxide dismutase, SOD2, by 83% (*P* <0.0001). Galactose treatment did not alter the abundance of any of the other anti-oxidant proteins. We further verified the reduction in SOD2 protein levels by Western blot (Figure 2E) and gene expression (Figure 2 F). We then examined the effect of galactose culture on the total activity of superoxide dismutase (tSOD) enzymes after 1 and 5 days of galactose culture. One day in galactose culture did not alter tSOD activity, and 5 days in galactose culture showed a trend for a reduction in tSOD activity (*P* = 0.07; Figure 2G). When the activity of SOD2 was specifically tested, we observed a 59% reduction in enzymatic activity after 5 days in galactose culture (*P* = 0.026; Figure 2H). Given that glutathione redox homeostasis was retained in galactose culture despite the significant reduction in SOD2 protein and activity, these findings suggest that reduced SOD2 capacity is coupled to a reduction in superoxide (O_2_^●−^) production. We tested this prediction by using a cell-permeable chemical spin-trap to quantify the rate of superoxide production by EPR after 1 day of glucose or galactose culture (Figure 3A). These results showed that 1 day of galactose culture reduced the rate of superoxide production by 15% (Figure 3B; *P* = 0.031). Thus, galactose-stimulated mitochondrial respiration reduced the rate of cellular superoxide production as well as the expression of the mitochondrial anti-oxidant enzyme SOD2.

### Galactose-induced mitochondrial respiration downregulates hypoxia-inducible factor 2α and its target genes

**Figure 4:**
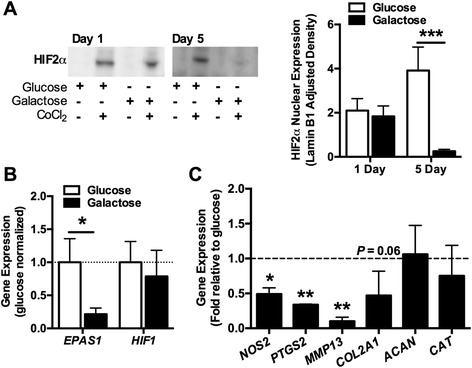
**Galactose-induced mitochondrial respiration reduces hypoxia-inducible factor 2α (HIF-2α) expression and signaling. (A)** Nuclear expression of HIF-2α expression was evaluated after 1 or 5 days of glucose versus galactose treatment. Twenty-four hours of cobalt chloride (CoCl_2_) treatment stabilized HIF-2α. Five days of galactose treatment dramatically reduced the nuclear expression of HIF-2α in CoCl_2_-stabilized samples (n = 3). **(B)** Five days of galactose treatment also reduced the expression of *EPAS1*, the gene that encodes HIF-2α, but not *HIF1* (n = 5). **(C)** The expression of multiple HIF-2α target genes, including *NOS2*, *PTGS2*, and *MMP13*, was also reduced after 5 days of a galactose culture. Results from galactose-treated chondrocytes were normalized to those from glucose samples to represent the fold induction in response to galactose. Bars represent mean ± standard error of the mean. *P <0.05, **P <0.01, and ***P <0.001 between glucose and galactose.

To better understand how a nutrient-induced shift toward mitochondrial respiration downregulates SOD2 expression, we investigated the expression of the transcription factor HIF-2α. HIF-2α regulates the transcription of genes that coordinate cellular metabolic and anti-oxidant responses during development and in response to metabolic and oxidative stresses, including *SOD2* [38]. After 1 day in culture with CoCl_2_, which stabilizes HIF-2α [31], we detected the nuclear expression of HIF-2α in both glucose- and galactose-fed cells (Figure 4A). However, after 5 days in galactose culture, HIF-2α nuclear expression was significantly reduced (Figure 4A). Gene expression of *EPAS1*, the gene that encodes HIF-2α, was also significantly downregulated after 5 days of galactose culture (Figure 4B). HIF-1α gene expression, however, was unchanged after 5 days of galactose culture (*P* = 0.64; Figure 4B).

HIF-2α transcriptionally regulates the expression of a number of pro-inflammatory and catabolic genes in chondrocytes, including *NOS2*, *PTGS2*, *MMP13*, and *ADAMTS4* [39,40]. After 5 days of galactose culture, the expression of *NOS2*, *PTGS2*, and *MMP13* was significantly reduced (Figure 4C), consistent with the downregulation in HIF-2α. *ADAMTS4* was detected in only two samples, although the fold reduction in expression relative to glucose was substantial in both samples (0.15 and 0.07). The expression of cartilage extracellular matrix proteins *COL2A1* and *ACAN* was not significantly altered with galactose culture; however, similar to the catabolic genes, *COL2A1* mRNA expression trended lower (Figure 4C). We also examined the expression of the anti-oxidant enzyme catalase (*CAT*), whose activity is significantly reduced in chondrocytes after HIF-2α small interfering RNA (siRNA) treatment [41]. In the current study, galactose-induced downregulation of HIF-2α was not associated with a reduction in *CAT* expression.

**Figure 5:**
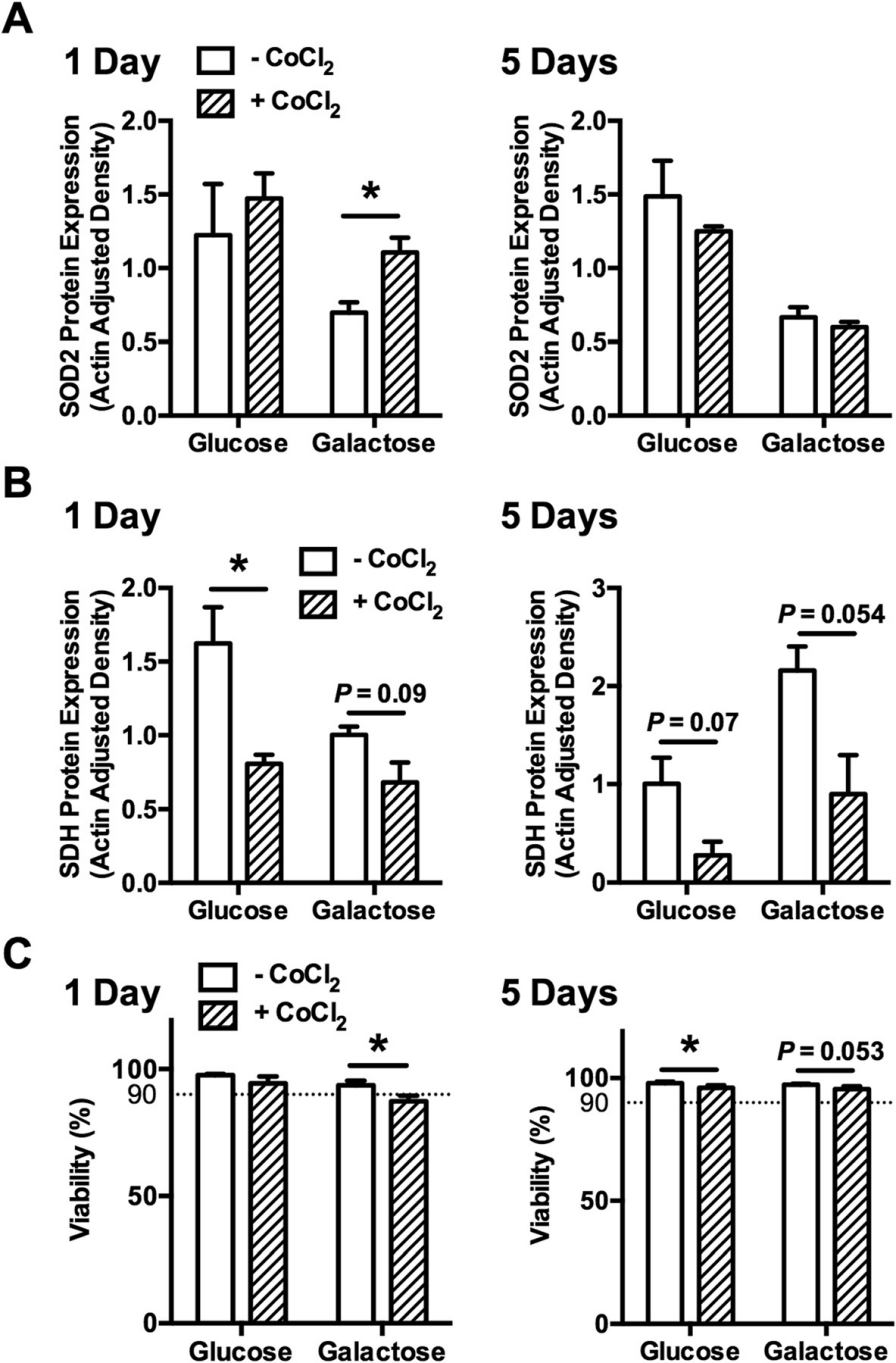
**Stabilization of hypoxia-inducible factor 2α (HIF-2α) impairs acute galactose-induced redox coupling and cell viability.** Chondrocytes were cultured in glucose- or galactose-supplemented media for 1 or 5 days and were treated with cobalt chloride (CoCl_2_) for the final 24 hours to evaluate the effect of acute stabilization of HIF-2α. **(A)** CoCl_2_-mediated HIF-2α stabilization after 1 day of culture prevented the galactose-induced reduction in superoxide dismutase 2 (SOD2) expression but had no effect on expression at 5 days or in any glucose culture condition (n = 3). **(B)** CoCl_2_ treatment reduced succinate dehydrogenase (SDH) expression in 1-day glucose cultured cells and showed a trend for reduced expression in galactose-treated samples at 1 and 5 days. Notably, CoCl_2_ treatment prevented the upregulation in SDH expression after 5 days of galactose culture (n = 3). **(C)** HIF-2α stabilization reduced cell viability in galactose- but not glucose-treated chondrocytes during the acute (1 day) response to galactose (n = 3). After 5 days in glucose or galactose culture, HIF-2α stabilization modestly reduced cell viability in glucose-treated cells, with a trend for reduced viability after galactose treatment (n = 6). Bars represent mean ± standard error of the mean. *P <0.05 between +/− CoCl_2_ treatment.

We subsequently investigated how stabilizing HIF-2α affected the galactose-induced changes in redox and metabolic coupling. CoCl_2_ was added to the culture media 24 hours prior to harvesting cells cultured for 1 or 5 days in galactose- or glucose-supplemented media. After 1 day in galactose media, SOD2 expression decreased by 43% compared with the glucose controls (Figure 5A). CoCl_2_ treatment blocked this reduction in SOD2 levels in galactose-cultured cells (*P* = 0.028) without altering those in glucose-supplemented media (*P* = 0.55). After 5 days in galactose media, SOD2 expression was not altered by CoCl_2_ treatment (Figure 5A). These data suggest that HIF-2α stabilization is sufficient to regulate the acute (1 day), but not the sustained (5 day), downregulation in SOD2 expression that occurs in response to upregulated mitochondrial respiration. We examined the effect of CoCl_2_ treatment on SDH expression to evaluate how stabilizing HIF-2α alters metabolic coupling. Unlike the effects on SOD2 expression, CoCl_2_ treatment primarily reduced the expression of SDH after 1 day of glucose culture, with a trend for reduced expression with galactose culture as well (Figure 4B). These findings suggest that HIF-2α is a negative regulator of SDH expression independent of galactose treatment. Interestingly, we observed that the effect of CoCl_2_ treatment on cell viability was reduced in chondrocytes cultured with galactose for 1 day but not 5 days (1-day viability: 93.7% ± 1.8% versus 87.3% ± 2.2%, − CoCl_2_ versus + CoCl_2_, *P* = 0.017; 5-day viability: 97.3% ± 0.4% versus 95.6% ± 1.1%; − CoCl_2_ versus + CoCl_2_; *P* = 0.053; Figure 5C). CoCl_2_ treatment did not alter cell viability in glucose culture at day 1 but caused a slight, albeit significant, reduction at day 5 (97.9% ± 0.7% versus 96.1% ± 1.0%; − CoCl_2_ versus + CoCl_2_; *P* = 0.025; Figure 5C). Thus, stabilizing HIF-2α expression reduces cell viability, and the greatest effect is observed during an acute increase in mitochondrial respiration.

## Discussion

Chondrocytes rely primarily on non-oxidative glycolysis to generate ATP for cellular energy [8,10,11]. Yet under conditions of glucose deprivation or glycolysis inhibition, chondrocytes increase oxygen consumption as a compensatory response to maintain ATP production via the MRC (that is, ‘the Crabtree effect’) [9,42]. The ability of chondrocytes to respond to changes in substrate availability by altering their reliance on glycolysis versus oxidative phosphorylation for ATP production is critical for cell survival and for maintaining extracellular matrix production [43,44]. However, the effect of this metabolic flexibility on other cellular functions, such as cellular oxidation and anti-oxidant defense pathways, is not well understood in chondrocytes.

In this study, we tested the ability of primary bovine chondrocytes to use oxidative phosphorylation to generate ATP and maintain cell viability using a nutrient trigger to upregulate mitochondrial respiration in mammalian cells. This trigger—galactose—induced a metabolic stress in chondrocytes, as indicated by a modest reduction in the cellular energy charge and an increase in the ratio of NAD^+^ to NADH. In response to this stress, chondrocytes increased their rate of oxygen consumption and upregulated the mitochondrial respiratory chain and Krebs cycle enzyme SDH. The increase in mitochondrial respiration did not alter the cellular redox balance, as indicated by a stable ratio of reduced to oxidized glutathione. However, galactose treatment did substantially reduce the production of nitric oxide, consistent with a negative relationship between mitochondrial respiration and nitric oxide production in chondrocytes [16,18,45]. Galactose treatment also significantly reduced the generation of superoxide (O_2_^●−^), a reactive molecule rapidly converted to hydrogen peroxide by the anti-oxidant enzyme superoxide dismutase (SOD). We found that the mitochondrial isoform of SOD, SOD2, was selectively reduced after galactose treatment. These findings show that stimulating chondrocyte mitochondrial respiration has a profound impact on the production and consumption of cellular ROS, which results in the maintenance of redox homeostasis.

The ability of a mitochondrial metabolic stimulus to induce substantial changes in SOD2 expression has important implications for understanding the origins of cartilage oxidative stress that occurs with aging and the development of OA. Several laboratories have reported that SOD2 expression is reduced in OA cartilage [8,19-21]. Gavriilidis and colleagues recently evaluated the association between a reduction in SOD2 expression and an increase in cartilage oxidation by depleting SOD2 in human articular chondrocytes using RNA interference [8]. They found that a loss of SOD2 induced lipid peroxidation and mitochondrial DNA strand breaks, verifying an inverse link between SOD2 levels and chondrocyte oxidation. Intriguingly, they also found that SOD2 depletion reduced the spare respiratory capacity and increased mitochondrial ATP turnover. Thus, the findings of this and our current study show an inverse relationship between mitochondrial respiration and SOD2 expression in chondrocytes.

This raises important questions about the extent to which metabolic stress signals contribute to OA risk by coupling the downregulation of SOD2 with increased mitochondrial respiration. OA cartilage is characterized by a reduction in the expression of several glycolytic enzymes and increased mitochondrial respiration under basal conditions [8,46]. Our finding that a nutrient-induced increase in mitochondrial respiration is coupled to a significant reduction in SOD2 expression may make chondrocytes more susceptible to oxidative damage. For example, well-established risk factors for OA, such as biomechanical trauma and increased levels of pro-inflammatory cytokines, damage chondrocytes in part by increasing the production of mitochondrial ROS [47-50]. Under acute stress, chondrocytes upregulate SOD2 expression to counteract increased ROS production via nuclear factor-kappa-B (NF-κB) signaling, which is the primary stress-responsive transcription factor regulating SOD2 expression [51,52]. Metabolic stress sensors, such as AMP-activated protein kinase (AMPK) [53] and sirtuins [54], are potent anti-inflammatory mediators, in part through inhibition of NF-κB signaling. Thus, it is not clear whether the downregulation of SOD2 resulting from a nutrient stress makes chondrocytes more susceptible to oxidative damage, because NF-κB activation can also promote ROS production in a feed-forward manner.

Liu-Bryan and Terkeltaub recently reviewed the dual roles of AMPK and SIRT1 in linking cellular bioenergetic sensing to the regulation of transcriptional ‘go signals’ (for example, NF-κB, HIF-2α, and MTF1) that initiate pro-inflammatory chondrocyte reprogramming [55]. In chondrocytes, activation of AMPK and SIRT1 attenuates the catabolic response to pro-inflammatory and biomechanical stressors [56-59]. In our study, sustained galactose feeding for 5 days would be expected to activate AMPK because of the reduction in cellular energy charge. Similarly, the increased ratio of NAD^+^ to NADH would be expected to drive the expression and activation of SIRT1. Consistent with an expected reduction in pro-inflammatory mediators, the expression of *NOS2* and *PTGS2* was significantly reduced after galactose culture. Our results suggest that a key pro-inflammatory ‘go signal’ that is downregulated in response to a nutrient stress is HIF-2α.

HIF-2α is a redox-sensitive and stress-responsive transcription factor. It transactivates multiple gene targets involved in cellular anti-oxidant defense, including SOD2 [38], and is also a central regulator of endochondral ossification and articular cartilage matrix homeostasis [39,40,60]. HIF-2α positively regulates the expression of genes that mediate chondrocyte hypertrophy, such as *COL10A1*, *MMP13*, and *VEGFA*. These actions of HIF-2α are recapitulated during the pathogenesis of OA, where HIF-2α promotes cartilage catabolism by upregulating the expression of pro-inflammatory and pro-hypertrophic genes in articular cartilage [39,40]. Although the stability of HIF-2α protein expression is regulated by oxygen-dependent hydroxylases, the activity of HIF-2α is regulated by post-translational reversible protein acetylation [61]. This suggests that HIF-2α is well suited to coordinate changes in cellular metabolic and redox status. Indeed, Bohensky and colleagues previously reported that silencing HIF-2α stimulated ROS production and activated a robust autophagic response [41]. Our findings show that stabilizing HIF-2α during an acute nutrient stress causes cell death, possibly by disrupting a pro-survival autophagic response. However, after 5 days of galactose-stimulated mitochondrial respiration, CoCl_2_ treatment did not reduce cell viability. This may be due to the downregulation in *EPAS1* expression, which could diminish the effectiveness of CoCl_2_ to activate HIF-2α-mediated signaling.

Our study provides the first observation that HIF-2α expression is negatively associated with increased mitochondrial metabolism in chondrocytes. This observation is important because it suggests that changes in cellular metabolism may directly mediate HIF-2α signaling. Elevated succinate levels promote HIFα stabilization by impairing oxygen-dependent prolyl hydroxylase activity, which targets HIFα for degradation [62,63]. In our study, galactose treatment significantly increased SDH expression in chondrocytes. An increase in the utilization of succinate to generate reducing equivalents for mitochondrial respiration would be expected to decrease succinate-mediated inhibition of prolyl hydroxylase activity, thereby promoting HIF-2α degradation. We also observed a decrease in *EPAS1* gene expression with galactose culture, indicating that stimulating mitochondrial respiration downregulates HIF-2α through additional genetic mechanisms.

Our findings build on recently reviewed work [55] suggesting that manipulating cellular metabolic stress responses may provide an integrative therapeutic approach to modify cartilage inflammation and degradation. Key questions remain, however, about how to implement such therapies and which molecules or pathways to specifically target. For example, it is not known whether a systemic metabolic stress, as occurs with weight loss or caloric restriction, is sufficient to induce a metabolic stress response in articular cartilage. Lifelong caloric restriction does not alter age-associated knee OA in 24-month old mice, but it does delay the development of hip OA in dogs [64,65]. Furthermore, our study suggests that downregulation of HIF-2α contributes to the anti-catabolic effects of a nutrient metabolic stress. However, sustained inhibition of HIF-2α may be deleterious as it also promotes cartilage anabolism [66]. Implementing metabolic-based therapies will likely require a more comprehensive understanding of the dynamic *in vivo* changes that occur in chondrocyte metabolic signaling and energetic flux during normal and pathologic conditions. Such knowledge may also lead to a better understanding of OA risk factors, such as with obesity-associated metabolic disease clustering and mitochondrial DNA haplogroup variants [67,68].

## Conclusions

Our findings show that chondrocyte metabolic flexibility promotes cell survival during periods of limited nutrient availability by upregulating mitochondrial respiration. The increase in mitochondrial metabolism is coupled to a substantial reduction in the expression and activity of the mitochondrial anti-oxidant SOD2 and its pro-catabolic transcription factor HIF-2α. Both SOD2 and HIF-2α undergo differential expression during the development of OA, initially being upregulated after acute inflammation and subsequently downregulated with disease progression [21,39-41,51]. Thus, an improved understanding of chondrocyte metabolism during OA pathogenesis may lead to new insight into the etiology of OA and the development of metabolic-based therapeutic targets.

## Abbreviations

AMPK: 5′ adenosine monophosphate-activated protein kinase
CAT: catalase
CMH: 1-hydroxy-3-methoxycarbonyl-2,2,5,5-tetramethylpyrrolidine
CoCl_2_: cobalt chloride
DMEM: Dulbecco’s modified Eagle’s medium
EPR: electron paramagnetic resonance
ESA: equine serum albumin
GSH: reduced glutathione
HIF-2α: hypoxia-inducible factor 2α
LDH: lactate dehydrogenase
MRC: mitochondrial respiratory chain
MSC: mesenchymal stem cell
NF-κB: nuclear factor-kappa-B
NO_x_: nitrate + nitrite
O_2_^●−^: superoxide
OA: osteoarthritis
PBS: phosphate-buffered saline
ROS: reactive oxygen species
SDH: succinate dehydrogenase
SOD: superoxide dismutase
SRM: selected reaction monitoring
tSOD: total activity of superoxide dismutase
